# Assessment of mental health problems among adolescents in Sri Lanka: Findings from the cross‐sectional Global School‐based Health Survey

**DOI:** 10.1002/hsr2.886

**Published:** 2022-10-17

**Authors:** Gajarishiyan Rasalingam, Arrosan Rajalingam, Miyuru Chandradasa, Mintu Nath

**Affiliations:** ^1^ Institute of Applied Health Sciences University of Aberdeen Aberdeen UK; ^2^ Institute of Medical Sciences University of Aberdeen Aberdeen UK; ^3^ Department of Psychiatry University of Kelaniya Ragama Sri Lanka

**Keywords:** anxiety, Global School‐based Student Health Survey, loneliness, mental health adolescents, suicidal ideation

## Abstract

**Background and Aims:**

Mental health condition among adolescents is a leading cause of health‐related disability in Sri Lanka. The study aims to estimate the prevalence and evaluate the associated risk factors in three major mental health domains—loneliness, anxiety and suicidal ideation—among Sri Lankan adolescents.

**Methods:**

We conducted a secondary analysis of cross‐sectional data of 3262 adolescents from the Global School‐based Health Survey (GSHS) conducted by the WHO in 2016. We modeled the binary outcome variables using multivariable logistic regression models with exposures representing demography, food habits, personal hygiene, behavior, substance abuse, parental and social engagement of the respondents.

**Results:**

We estimated the prevalence of loneliness, anxiety and suicidal ideation as 30.8% (95% CI: 29.3, 32.5), 20.2% (95% CI: 18.8, 21.6) and 3.7% (95% CI: 3.1, 4.4), respectively, and the overall prevalence as 40.3% (95% CI: 38.6, 42.0). Mental health problems were more prevalent among females than males. Engagement with parents and close friends, adequate nutritional intake and physically active lifestyles reduced the risk of common mental health problems. Exposure variables like food insecurity, truancy, second‐hand smoking, physical fight, and being bullied increased adolescents’ risk of reported psychological problems.

**Conclusions:**

We conclude that the prevalence of mental health problems in the Sri Lankan adolescent population was higher than the global average. Results suggest that future policy decisions to mitigate mental health problems among Sri Lankan adolescents should incorporate an integrated approach involving the individual, family and community to promote positive home and school environments combined with an active and healthy lifestyle.

## 1 INTRODUCTION

Worldwide, 1.2 billion adolescents (between the ages of 10–19 years) represent 16 percent of the world population.^1^ Adolescence is an important physical, social, and cognitive transition period that translates to skills and opportunities influencing adult life.^2^ However, they are also exposed to uncertainties and risk factors, including noncommunicable diseases and mental health. As a result, the United Nations 2030 global strategy on “Every woman and every child” prioritized adolescents and their mental health well‐being.^3^

The World Health Organization (WHO) defined mental health as “*a state of well‐being in which the individual realizes his or her own abilities, can cope with the normal stresses of life, can work productively and fruitfully, and is able to make a contribution to his or her community*”.^4^ Approximately 10%–20% of adolescents worldwide suffer from mental health problems, a leading cause of health‐related disability.^5^ Mental health problems should be addressed urgently in lower‐middle‐income countries (LMIC) that represent approximately 90% of the world's adolescent population.^1^ Practical implementations and scaling up of mental health services are still lagging in these countries due to the lack of policy decisions, limited commitment, and funding in health sectors.^5^, ^6^

Sri Lanka, an LMIC with a population of 22 million, of which one‐fifth are adolescents, has a reputed health infrastructure compared to its regional counterparts, but noncommunicable diseases like mental health and malnutrition remain a challenge. Based on limited data available from outpatient child and adolescent mental health services^7^ and school‐going children,^8^ an increased prevalence of the mental health status among adolescents in Sri Lanka is a growing concern. However, the data on the prevalence of mental health conditions, particularly among adolescents in Sri Lanka, is still unknown.^9^ The Sri Lankan health authorities have acknowledged the alarming nature of general mental health, including the higher suicide rates, increasing substance abuse, and psychosocial problems in children and adolescents.^10^

Mental well‐being is a broad and complex topic. Some researchers specifically considered three major mental health domains—loneliness, anxiety, and suicidal ideation—to measure the psychological status of the adolescent in line with standard mental health directories.^11^, ^12^ The World Health Organization (WHO) and Centres for Disease Control and Prevention (CDC), in collaboration with other United Nations agencies, developed and promoted the Global School‐based Health Survey (GSHS). The GSHS data provide an excellent resource for understanding adolescents' physical and mental well‐being on multiple factors, including dietary habits, hygiene, behavioral, substance abuse, injuries, and protective factors. Given the multifaceted factors contributing to mental health problems, the present study aims to summarize the GSHS data and provide recommendations to support health professionals and policymakers in addressing the mental health issues among adolescents in Sri Lanka. We set out two objectives: first, to estimate the prevalence of mental health issues in the adolescent population in Sri Lanka, and second, to evaluate the factors associated with mental health issues in this population.

## 2 METHODS

### 2.1 Study design

This study is a secondary analysis of cross‐sectional data from the GSHS conducted in Sri Lanka in 2016.

### 2.2 Data source

The GSHS is a school‐based health survey completed through a self‐administrated questionnaire by adolescents in grades 8–13. In collaboration with other United Nations agencies, the WHO and the CDC jointly developed the survey to measure the healthy behavior and protective factors among pupils attending school. The GSHS survey data are publicly available online on the WHO website (https://www.who.int/ncds/surveillance/gshs/en/).

### 2.3 Data collection procedure

The GSHS survey data were collected using a two‐stage clustering method to select the representative of the study population. In the first stage, 40 schools were selected based on the probability proportional to the number of enrollments in grades 8–13 (Supporting Information: Figure S1). In the second stage, grades 8–13 classrooms were randomly chosen, and each student in the selected classroom was eligible to participate. To increase the likelihood of sampling and to reduce the bias of the nonresponses to the survey, an appropriate weighting factor was used to validate the study.^13^ The Ethics Review Committee of the Faculty of Medicine, the University of Colombo, approved the study. The questionnaire was prepared in three main languages (Sinhala, Tamil, and English) widely spoken in Sri Lanka. All students provided their consent, and appropriate measures were taken regarding the confidentiality of participants and contamination of responses. All enrolled schools participated, and 89% of sampled students responded.

### 2.4 Study variables

Based on 58 questions measured in the Sri Lanka GSHS study, we constructed seven broad domains with relevant predictor variables: demography, food habits, personal hygiene, behavioral, substance abuse, parental, and social engagement. One question was used to identify the response variables loneliness (Q22), anxiety (Q23), and two questions (Q24 and Q25) to derive the variable suicidal ideation. Details of the predictor variables within each domain and data recoding strategies are presented in Supporting Information: Table S1.

### 2.5 Statistical analysis

We presented the descriptive statistics of all study variables using the total frequency and percentage. We estimated the prevalence of loneliness, anxiety, suicidal ideation and corresponding 95% confidence interval using the Wilson procedure with continuity correction. We fitted separate logistic regression models in a single (unadjusted) and multivariable (adjusted) framework to evaluate the association of different predictor variables. Predictor variables of the model included variables that correspond to demography, food habits, personal hygiene, behavioral, substance abuse, parental, and social engagement. The multivariable model selection between the competitive models was assessed using the Akaike information criterion (AIC) and the likelihood ratio test statistic. All statistical tests were two‐sided with the type 1 error rate set at 5%. We obtained the estimates of odds ratio (OR) and corresponding 95% confidential interval (CI) from the final fitted models. All statistical analyses were conducted in the R software environment (version 3.6.0).

## 3 RESULTS

### 3.1 Prevalence of mental health problems

**Figure 1:**
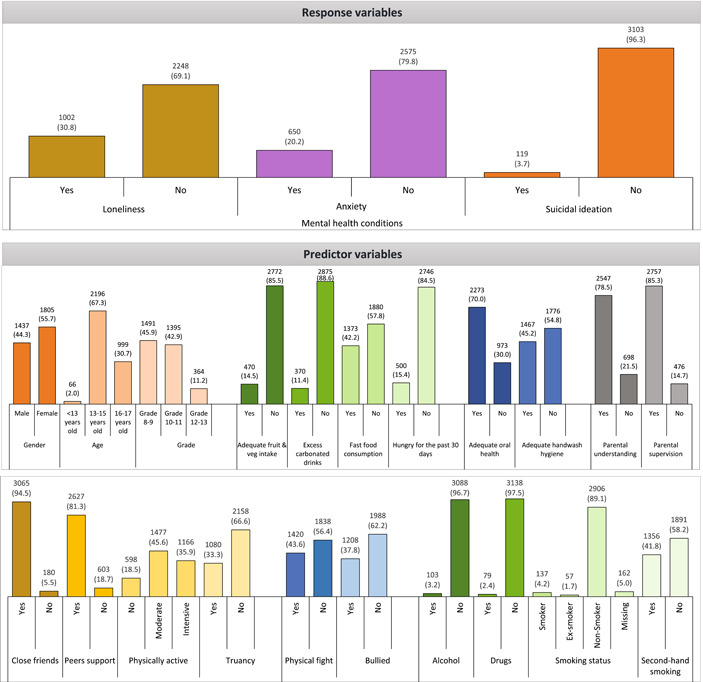
Summary statistics of different categories of the response and predictor variables (frequency, percentage in parenthesis) measured on the Global School‐based Health Survey data

A total of 3262 school‐going adolescents in Grade 8–13 participated in the survey. Figure 1 presents the summary statistics of demography, behavioral, and psychosocial characteristics. The survey recorded that 40.3% (95% CI: 38.6, 42.0) of respondents experienced some form of mental health problem 1 year before the survey. Among them, 30.8% (95% CI: 29.3, 32.5) perceived loneliness, 20.2% (95% CI: 18.8, 21.6) experienced anxiety and 3.7% (95% CI: 3.1, 4.4) reported suicidal ideation.

### 3.2 Summary of predictor variables

The survey received slightly higher responses from females (55.7%, *n* = 1805) than males (44.3%, *n* = 1437). Most respondents (85.5%, *n* = 2772) did not consume adequate fruit and vegetable, and more than 40% of adolescents consumed fast food (42.2%, *n* = 1373), while 15.4% of households had identifiable food insecurity. Approximately half of the population practised good hand hygiene, and nearly 70% of adolescents had adequate oral hygiene. According to responses, approximately 80% of respondents received good parental and social support. Among the behavioral characteristics, 43.6% (*n* = 1420) engaged in a physical fight, and 37.8% (*n* = 1208) reported the experience of being bullied. More than 40% of respondents were exposed to second‐hand smoking (41.8%, *n* = 1356), and a smaller percentage of the respondents had psychoactive substance abuse like cigarette smoking (4.2%, *n* = 137), alcohol (3.2%, *n* = 103), and cannabis (2.4%, *n* = 79).

### 3.3 Factors associated with loneliness

**Figure 2:**
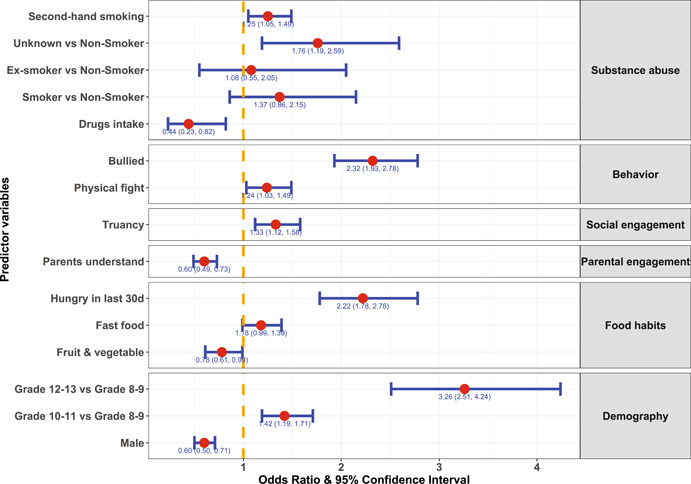
Estimates of the odds ratio and corresponding 95% confidence interval of categories of predictors (compared with the reference category) from the fitted multivariable logistic regression model of loneliness

The outcomes from the fitted logistic regression model of loneliness and its association with different predictor variables are presented in Supporting Information: Table S2 and summarized in Figure 2. The outcomes from the adjusted model showed that females, students in higher grades, and those who consumed inadequate amounts of fruits and vegetables or were hungry during the past 30 days had an increased likelihood of loneliness. Among behavioral, parental, and social engagement predictors, adolescents exposed to second‐hand smoking, experienced reduced parental support, were involved in truancy, engaged in a physical fight, and were bullied suffered increased loneliness.

### 3.4 Factors associated with anxiety

**Figure 3:**
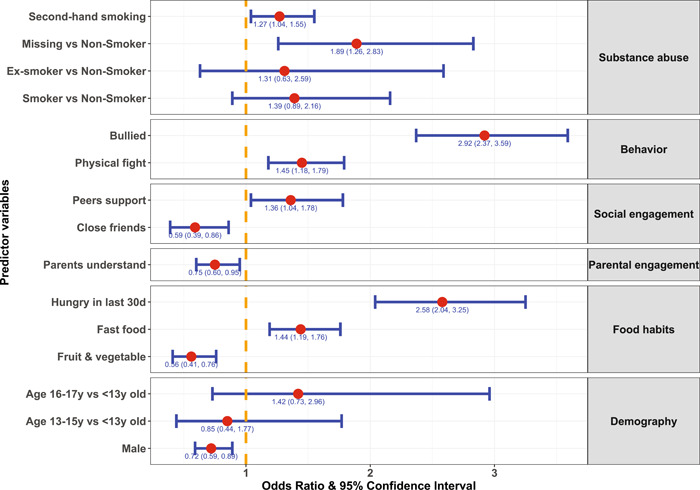
Estimates of the odds ratio and corresponding 95% confidence interval of categories of predictors (compared with the reference category) from the fitted multivariable logistic regression model of anxiety

Supporting Information: Table S3 and Figure 3 present the outcomes from the logistic regression model of anxiety. Like loneliness, the odds of anxiety were higher among female adolescents who studied in higher grades, consumed fast foods, took inadequate fruits and vegetables, or were hungry for 30 days. Similarly, adolescents who perceived reduced parental understanding, lack of close friends, second‐hand smoking or engaged in a physical fight or being bullied had an increased level of anxiety. Interestingly, adolescents with increased peer support also had an increased anxiety level.

### 3.5 Factors associated with suicidal ideation

**Figure 4:**
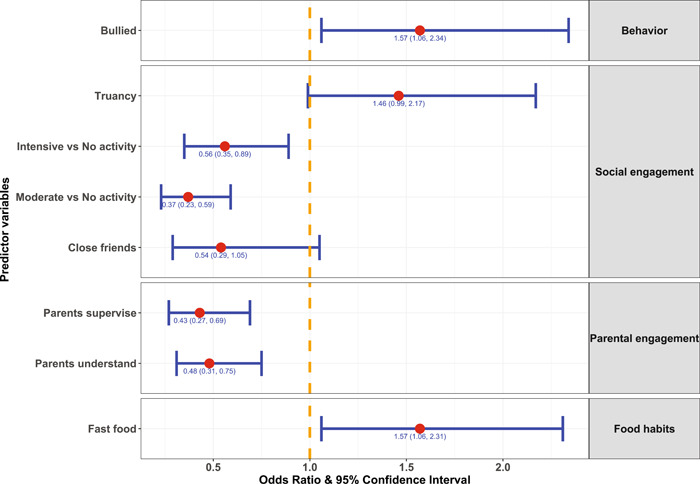
Estimates of the odds ratio and corresponding 95% confidence interval of categories of predictors (compared with the reference category) from the fitted multivariable logistic regression model of suicidal ideation

Supporting Information: Table S4 and Figure 4 present the outcomes from the logistic regression model of suicidal ideation. Increased parental and social engagement through perceived parental understanding, parental supervision, and close friends were associated with less reported suicidal ideation. Adolescents engaged in physical activity had decreased suicidal thoughts compared to those who were not physically active. The experience of bullying increased the odds of suicidal thoughts.

## 4 DISCUSSION

Using one of the largest survey data on the adolescent population in Sri Lanka, the present study assessed the prevalence of mental health problems among school students between grades 8–13 and evaluated the factors associated with their psychological health. The prevalence estimates of loneliness, anxiety and suicidal ideation were 30.8%, 20.2%, and 3.7%, respectively, while the overall prevalence (%) was 40.3 (95% CI: 38.6, 42.0). We observed increased engagement with parents and close friends, adequate nutritional intake and physically active lifestyles reduced the likelihood of mental health problems. On the other hand, food insecurity, truancy, exposure to second‐hand smoking, physical fight, and being bullied increased the odds of mental health problems among Sri Lankan adolescents.

The overall prevalence (40.3%) of mental health problems among adolescents in Sri Lanka is higher than the reported global average estimate of prevalence of 10%–20% in the adolescent and children population.^5^ In a recent school survey, conducted in four lower‐ and middle‐income countries, including Sri Lanka, the reported prevalence of loneliness among the local participants was 9%, anxiety at 5.5%, and suicidal ideation at 7.9%.^14^ The items in the questionnaire used in this survey were derived and modified from various sources, including GSHS. We also observed that mental health problems were more prevalent among female pupils confirming that girls are affected more than boys and seek more help for mental health problems.^15^ Many factors contribute to women's mental health and well‐being, such as violence, socioeconomic disadvantage, inequalities, social status, and unremitting caring responsibilities.^16^ As observed in the current study, an increased likelihood of loneliness and anxiety among upper‐grade students has been reported earlier^17^; this could be due to the critical phase of grade 12–13 students who prepare for various competitive examinations and university admissions. A study assessing a similar age group of upper‐grade students in Sri Lanka revealed higher mental health problems with 28% anxiety and 36% reported depression, and significantly more symptoms in female pupils.^17^

A large percentage (85%, *n* = 2772) of adolescents had lower nutritional supplements, particularly inadequate consumption of fruits and vegetables. The WHO and World Food Program recommend a daily dietary intake of at least 400–500 g per day, about two servings of fruit and three servings of vegetables.^18^ Nutritional problems are common worldwide; less than 30 percent of adolescents meet the WHO nutrition requirement.^19^ The relationship between consuming fruits and vegetables and mental health remains unclear, but studies confirmed the positive association between subjective mental health well‐being and adequate intake of fruits and vegetables.^20^ This study also found that adolescents who experienced hunger were more likely to experience loneliness and anxiety. It is suggested that the lower social‐economic status of the family may lead to food insecurity and hunger, which may negatively impact physical and mental well‐being including substance abuse, anxiety, and behavioral problems.^21^

The study demonstrates that moderate or intensive physical activity had a beneficial effect on alleviating mental health problems, especially with suicidal ideation. Many cross‐sectional studies confirmed the positive impact of physical activity on mental health. Several plausible mechanisms are proposed to explain such association: physical activity acts as an emotional buffer against stressful events,^22^ increases self‐esteem,^23^ and distracts from negative thoughts^24^; and possibly regular exercise increases the release of the neurotransmitters such as dopamine, norepinephrine, and serotonin, involved in mood regulation.^25^

The positive influence of parental engagement, as observed in this study, is consistent with the previous studies^26^; parental understanding reduces the odds of experiences of loneliness, anxiety, and suicidal ideation. A similar study in Sri Lanka has found that a large percentage of children experience psychological and physical violence at home and school, with a lifetime prevalence of 46% corporal punishment by parents and 80.4% during a school term.^27^ This probably signifies that many parents in Sri Lanka are involved with children but need better strategies and support to deal with the behavioral problems of the children.

This study found that bullied adolescents were more vulnerable as they suffered severe mental health problems, including suicidal ideation. Bullying is the most common form of social evil faced by adolescents, particularly in school surroundings.^28^ On the other hand, access to close friends and good school attendance contributed positively and reduced the odds of feeling lonely and anxious. Enhanced parental involvement, good dietary habits and increased physical activity were recognized to improve mental health problems among adolescents.^29^ Similar adverse childhood experiences regarding emotional neglect and witnessing community violence were reported among school‐going adolescents in Tunisia.^30^ Although the present study did not assess the interaction of these factors explicitly, other studies documented that the interaction of these factors reduced the incidences of bullying.^10^ Notwithstanding, it is evident from this study that parent‐child engagement and understanding are conducive to good healthy growth and personality development. Parents can play a pivotal role in encouraging and offering advice on better food habits, increased personal hygiene, and appropriate physical activity to make the adolescent period more supportive.

We identified that over 40% of the adolescents in this study were exposed to second‐hand smoking. Globally, adolescents are more exposed to second‐hand smoking than other age groups.^31^ Second‐hand smoking was associated with an increased likelihood of experiencing loneliness and anxiety. Similar findings are also supported in the literature.^32^, ^33^ The possible association of second‐hand smoking with increased mental health issues may be manifested due to several reasons: physical discomfort due to the toxic agents^34^; the adverse influence on the neurotransmitters due to increased nicotine level in the blood,^35^ and positive association between second‐hand smoking and chronic health conditions such as respiratory diseases and obesity.^36^ This study revealed that adolescents who took psychoactive substances had a reduced likelihood of feeling lonely; it may be assumed that this is likely to be a short‐lasting effect, and there is evidence that self‐isolation leading to loneliness could increase the use of cannabis.^37^

Mental health conditions in adolescents are complex and multifaceted. Although this study considered only three domains of mental health of adolescents—loneliness, anxiety and suicidal ideation—it demonstrates pertinent findings that could be easily implementable as policy measures. To tackle mental health issues among adolescents, the parents, schools, and community should collaborate and work together to build an inclusive and integrated school curriculum. The collaboration should promote positive home and school environments with zero tolerance to bullying and encourage an active and healthy lifestyle with healthy eating patterns. The mental health problems among adolescents should be addressed at the individual, family, and community levels with planned and targeted school and community‐based awareness programs and regular screening and health promotion events. An integrated approach would help to identify and resolve these issues at the earliest and reduce their impact on the future adult population.

### 4.1 Study limitations

The present study has several limitations. First, the survey was conducted in 2016, therefore, it may not reflect the country's most recent mental health status, particularly during the pandemic. However, we believe some of the strategies outlined in this study could still be relevant. Second, it is estimated that 1–3 children are out of school in lower and middle‐income countries,^38^ and in Sri Lanka, 55% of the disabled adolescent population is out of school. Hence, this study may not generalize to the adolescent population representing the disabled, school dropouts, and school absentees. This study is less representative of the north and east provinces of Sri Lanka that experienced 30 years of civil war, resulting in higher incidences of disability and mental health trauma. Due to the self‐administrated nature of the questionnaire, the responses may also be under or over‐reported. The survey captured only selected features of mental health factors; hence an overall assessment of possible confounders at the individual, family, and community levels was not possible. To evaluate the scale of mental health problems in the current scenario, we recommend a comprehensive nationwide survey with appropriate survey weights based on the present adolescent population in Sri Lanka.

## 5 CONCLUSIONS

Using comprehensive nationwide survey data, we observed that 40% of adolescents in Sri Lanka suffered from mental health problems related to loneliness, anxiety or suicidal ideation; the estimated prevalence was more than double compared with the global average of 10%–20%. Increased engagement with parents and close friends, adequate nutritional intake and physically active lifestyles reduced the likelihood of common mental health problems. On the other hand, food insecurity, truancy, exposure to second‐hand smoking, engaging in a physical fight and being bullied increased the odds of adolescent mental health problems. Results suggest that future policy decisions to mitigate mental health problems among Sri Lankan adolescents should incorporate an integrated approach involving the individual, family, and community to promote positive home and school environments combined with an active and healthy lifestyle.

## AUTHOR CONTRIBUTIONS

**Gajarishiyan Rasalingam**: Conceptualization; data curation; formal analysis; investigation; methodology; resources; writing – original draft; writing – review and editing. **Arrosan Rajalingam**: Data curation; investigation; resources; writing – review and editing. **Miyuru Chandradasa**: Investigation; resources; writing – review and editing. **Mintu Nath**: Conceptualization; data curation; formal analysis; investigation; methodology; resources; writing – original draft; writing – review and editing. All authors have read and approved the final version of the manuscript.

## CONFLICT OF INTEREST

The authors declare no conflict of interest.

## TRANSPARENCY STATEMENT

The lead author Mintu Nath affirms that this manuscript is an honest, accurate, and transparent account of the study being reported; that no important aspects of the study have been omitted; and that any discrepancies from the study as planned (and, if relevant, registered) have been explained.

## Supporting information

Supporting information.

## Data Availability

The corresponding author had full access to the data used in this study and takes complete responsibility for the integrity of the data and the accuracy of the data analysis. The GSHS survey data are publicly available online on the WHO website. Data are anonymized to protect individual participant's identity. The data from Sri Lanka used in this study can also be accessed from the site. Web link: https://www.who.int/ncds/surveillance/gshs/en/. Further details of the data policy and relevant procedures are documented in the following weblink: https://www.cdc.gov/gshs/background/pdf/2005datapolicy.pdf
