# Supplementary material for: Assessment of mental health problems among adolescents in Sri Lanka: Findings from the cross‐sectional Global School‐based Health Survey
Source: Health Sci Rep. 2022 Oct 17;5(6):e886. doi: 10.1002/hsr2.886 (PMC9576112; doi:10.1002/hsr2.886)
Supplement: Supplementary file 1 — Supporting information. [file HSR2-5-e886-s001.pdf]

# Assessment of mental health conditions among adolescents in Sri Lanka: Findings from the cross-sectional Global School-based Health Survey

Gajarishiyan Rasalingam[1], Arrosan Rajalingam[2], Miyuru Chandradasa[3] and Mintu Nath[1*]

[1]Institute of Applied Health Sciences, University of Aberdeen, Aberdeen, AB25 2ZD, United Kingdom

[2]Institute of Medical Health Sciences, University of Aberdeen, Aberdeen, AB25 2ZD, United Kingdom

[3]Department of Psychiatry, University of Kelaniya, Ragama, Sri Lanka

## Contents

Supplementary Figure S1: Distribution of schools across the country selected for the Global School-based Health Survey. ........ 2

Supplementary Table S1: The variables used for the study from the Global School-based Health Survey (GSHS) questionnaire§ and data recording strategy. ........ 3

Supplementary Table S2: Estimates of odds ratio (OR) and corresponding 95% confidence interval (95% CI) from unadjusted (single variable) and adjusted (multivariable) logistic regression models of loneliness. ........ 5

Supplementary Table S3: Estimates of odds ratio (OR) and corresponding 95% confidence interval (95% CI) based on the unadjusted (single variable) and adjusted (multivariable) logistic regression models of anxiety. ........ 7

Supplementary Table S4: Estimates of odds ratio (OR) and corresponding 95% confidence interval (95% CI) based on the unadjusted (single variable) and adjusted (multivariable) logistic regression models of suicidal ideation. ........ 9

**Supplementary Figure S1: Distribution of schools across the country selected for the Global School-based Health Survey.**

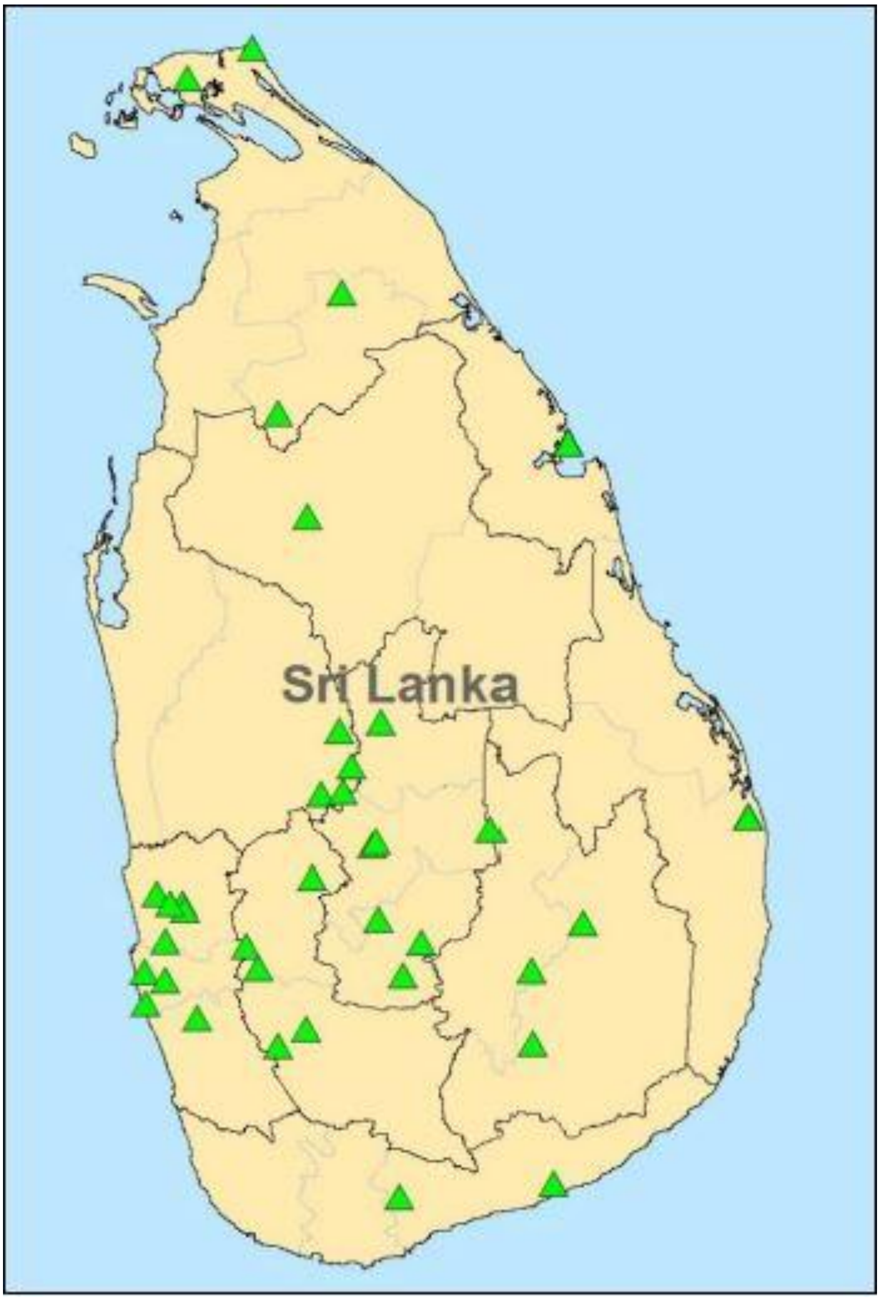


The sampling was conducted using two-stage clustering method. In first stage, schools were selected and in second stage, the classrooms are sampled within the selected school. Source: Ministry of Health, Nutrition and Indigenous Medicine, Sri Lanka

**Supplementary Table S1: The variables used for the study from the Global School-based Health Survey (GSHS) questionnaire[§] and data recording strategy.**

| Variable | (Question No.) Survey Question | Coding |
|---|---|---|
| **Predictor Variables** | | |
| **Demography** | | |
| Gender | (Q2) [a] What is your sex? | 0= Female 1= Male |
| Age | (Q1) How old are you? | 0= 12 years or younger<br>1= 13 and 15years old<br>2= 16 years or older |
| Grade | (Q3) In what grade are you? | 0= Grade 8 and 9<br>1= Grade 10 and 11<br>2= Grade 12 and 13 |
| **Food Habits** | | |
| Adequate fruit & vegetable intake [b] | (Q7) During the past 30 days, how many times per day you usually eat fruit?<br>(Q8) During the past 30 days, how many times per day you usually eat vegetable? | 0 = less than 2 times<br>1 = 2 or more times<br>0 = less than 3 times<br>1 = 3 or more times |
| Carbonated drink consumption | (Q9) During the past 30 days, how many times per day did you usually drink carbonated drinks? | 0 = less than 2 times<br>1 = 2 or more times |
| Fast food intake | (Q10) During the past 7 days, on how many days did you eat food from a fast-food restaurant? | 0 = 0 days<br>1 = 1 or more days |
| Food insecurity | (Q6) During the past 30 days, how often did you go hungry because there was not enough food in your home? | 0 = Never and Rarely<br>1 = Sometimes, Most of the time and Always |
| **Personal Hygiene** | | |
| Oral hygiene | (Q11) During the past 30 days how many times per day did you usually clean or brush your teeth? | 0 = less than 2 times<br>1 = 2 or more times |
| Hand hygiene [c] | (Q12, 13 & 14) During the past 30 days how often did you wash your hands before eating; toilet or latrine; how often did you use soap when washing hands | 0 = Never, Rarely, Sometimes, Most of the time<br>1 = Always |
| **Parental Engagement** | | |
| Parental understanding | (Q56) During the past 30 days, how often did your parents or guardian understand your problems and worries? | 0 = Never and Rarely<br>1 = Sometimes, Most of the time and Always |
| Parental supervision | (Q57) During the past 30 days, how often did your parents or guardian really know what you were doing with your free time? | 0 = Never and Rarely<br>1 = Sometimes, Most of the time and Always |
| **Social Engagement** | | |
| Close friends | (Q27) How many close friends do you have? | 0 = 0 friends<br>1 = 1, 2, 3 or more friends |
| Peers support | (Q54) During the past 30 days, how often were most of the students in your school kind and helpful | 0 = Never and Rarely<br>1 = Sometimes, Most of the time and Always |
| Physically active | (Q49) During the past 7 days, on how many days were you physically active for total of at least 60 | 0 = 0 days<br>1 = 1, 2, 3 days (Moderate) |

| | | |
|---|---|---|
| | minutes per day? Add up all the time you spent in any kind of physical activity each day) | 2 = 4, 5, 6 days (Intensive) |
| Truancy | (Q53) During the past 30 days, on how many days did you miss classes or school without permission | 0 = 0 days<br>1 = 1 or more days |
| **Behavior** | | |
| Physical fight | (Q16) During the past 12 months, how many times were you in a physical fight | 0 = 0 times<br>1 = 1 or more times |
| Bullied | (Q20) During the past 30 days on how many days were you bullied? | 0 = 0 times<br>1 = 1 or more times |
| **Substance Abuses** | | |
| Current alcohol consumption | (Q53) During the past 30 days, on how many days did you have a least one drink containing alcohol? | 0 = 0 days<br>1 = 1 or more days |
| Drugs intake | (Q42) During the past 30 days, how many times have you used marijuana? | 0 = 0 times<br>1 = 1 or more time |
| Smoking status [d] | (Q28) How old were you when you first tried a cigarette?<br><br>(Q29) During the past 30 days, on how many days did you smoke cigarette?<br>(Q31) During the past 12 months, have you ever tried to stop smoking cigarettes? | 0 = Smoker<br>1= Ex-Smoker<br>2 = Non-Smoker<br>3 = Unknown* |
| Secondhand smoking | (Q32) During the last 7 days, on how many days have people smoked in your presence | 0= No Secondhand smoker<br>1= Secondhand smoker |
| **Response Variable – Mental Health** | | |
| Loneliness | (Q22) During the past 12 months, how often have you felt lonely | 0 = Never and Rarely<br>1 = Sometimes, Most of the time and Always |
| Anxiety | (Q23) During the past 12 months, how often have you been so worried about something that you could not sleep at night | 0 = Never and Rarely<br>1 = Sometimes, Most of the time and Always |
| Suicidal ideation [e] | (Q24) During the past 12 months, did you ever seriously consider attempting suicide | 0 = No<br>1 = Yes |
| | (Q25) During the past 12 months did you make a plan how you would attempt suicide | 0 = No<br>1 = Yes |

[§]The standard questionnaire of the Global School-based Health Survey (GSHS) is available from the following web link:
https://extranet.who.int/ncdsmicrodata/index.php/catalog/648/related_materials

* The smoking status of these participants cannot be determined based on their responses
[a] Question reference to the questionnaire.
[b] Adequate vegetable and fruit intake is a combination of two questions Q7 & Q8, final is coded as 0 or 1, where 0= fruit (No) vegetable (No) & 1= fruit (Yes) vegetable (Yes)
[C] Hand hygiene is a combination of three questions Q13, Q14 & Q15, final is coded as 0 or 1, where 0= in all three questions (Never, Rarely, Sometimes, Most of the time) & 1= in all three questions (Always)
[d] Smoking status is determined by combination of three questions Q29, Q30 & Q31, final coded as smoker, Ex-smoker, non-smoker and unknown
[e] Suicidal ideation is a combination of two question Q24 & Q25, final coded as 0 or 1, where 0= No in both question and 1= yes in both question

**Supplementary Table S2: Estimates of odds ratio (OR) and corresponding 95% confidence interval (95% CI) from unadjusted (single variable) and adjusted (multivariable) logistic regression models of loneliness.**

| Variable | | Loneliness | | | |
|---|---|---|---|---|---|
| | | Unadjusted | | Adjusted | |
| | | OR (95% CI) | P-value | OR (95% CI) | P-value |
| **Demography** | | | | | |
| Gender | | | | | |
| | Female | 1 | | 1 | |
| | Male | 0.87 (0.74-1.01) | 0.061 | 0.60 (0.50-0.71) | <0.001 |
| Age | | | | | |
| | <13 years old | 1 | | | |
| | 13-15 years old | 1.17 (0.69-2.17) | 0.551 | | |
| | 16-17 years old | 1.94 (1.12-3.57) | 0.024 | | |
| Grade | | | | | |
| | Grade 8-9 | 1 | | 1 | |
| | Grade 10-11 | 1.25 (1.06-1.47) | 0.007 | 1.42 (1.19-1.71) | 0.001 |
| | Grade 12-13 | 2.70 (2.13-3.42) | <0.001 | 3.26 (2.51-4.24) | <0.001 |
| **Food Habits** | | | | | |
| Adequate fruit & veg intake | | | | | |
| | No | 1 | | 1 | |
| | Yes | 0.78 (0.63-0.97) | 0.028 | 0.78 (0.61-0.99) | 0.044 |
| Excess carbonate drinks | | | | | |
| | No | 1 | | | |
| | Yes | 0.94 (0.74-1.19) | 0.613 | | |
| Fast food consumption | | | | | |
| | No | 1 | | 1 | |
| | Yes | 1.31 (1.13-1.52) | 0.001 | 1.18 (0.99-1.39) | 0.053 |
| Hungry for the past 30 days | | | | | |
| | No | 1 | | 1 | |
| | Yes | 2.89 (2.37-3.50) | <0.001 | 2.22 (1.78-2.78) | <0.001 |
| **Personal Hygiene** | | | | | |
| Adequate oral health | | | | | |
| | No | 1 | | | |
| | Yes | 0.80 (0.68-0.94) | 0.005 | | |
| Adequate handwash hygiene | | | | | |
| | No | 1 | | | |
| | Yes | 0.78 (0.67-0.90) | 0.001 | | |
| **Parental Engagement** | | | | | |
| Parental understanding | | | | | |
| | No | 1 | | 1 | |
| | Yes | 0.58 (0.48-0.69) | <0.001 | 0.60 (0.49-0.73) | <0.001 |
| Parental supervision | | | | | |
| | No | 1 | | | |
| | Yes | 0.65 (0.53-0.79) | <0.001 | | |
| **Social Engagement** | | | | | |
| Close friends | | | | | |
| | No | 1 | | | |
| | Yes | 0.66 (0.48-0.90) | 0.008 | | |
| Peers support | | | | | |
| | No | 1 | | | |
| | Yes | 1.07 (0.88-1.30) | 0.497 | | |
| Physically active | | | | | |

| | | | | | |
|---|---|---|---|---|---|
| | No Active | 1 | | | |
| | Moderate Active | 0.74(0.61-0.91) | 0.003 | | |
| | Intensive Active | 0.79 (0.64-0.97) | 0.025 | | |
| Truancy | | | | | |
| | No | 1 | | 1 | |
| | Yes | 1.70 (1.45-1.98) | <0.001 | 1.33 (1.12-1.58) | 0.001 |
| **Behavior** | | | | | |
| Physical fight | | | | | |
| | No | 1 | | 1 | |
| | Yes | 1.68 (1.44-1.95) | <0.001 | 1.24 (1.03-1.49) | 0.020 |
| Bullied | | | | | |
| | No | 1 | | 1 | |
| | Yes | 2.72 (2.33-3.18) | <0.001 | 2.32 (1.93-2.78) | <0.001 |
| **Substance abuse** | | | | | |
| Alcohol | | | | | |
| | No | 1 | | | |
| | Yes | 1.85 (1.24-2.76) | 0.002 | | |
| Drugs | | | | | |
| | No | 1 | | 1 | |
| | Yes | 1.38 (0.86-2.19) | 0.178 | 0.44 (0.23-0.82) | 0.011 |
| Smoking status | | | | | |
| | Non-Smoker | 1 | | | |
| | Smoker | 1.78 (1.25-2.52) | 0.001 | 1.37 (0.86-2.15) | 0.182 |
| | Ex-Smoker | 1.23 (0.69-2.12) | 0.467 | 1.08 (0.55–2.05) | 0.825 |
| | Unknown | 1.94 (1.40-2.67) | <0.001 | 1.76 (1.19-2.59) | 0.004 |
| Second-hand smoking | | | | | |
| | No | 1 | | 1 | |
| | Yes | 1.67 (1.43-1.93) | <0.001 | 1.25 (1.05-1.49) | 0.010 |

**Supplementary Table S3: Estimates of odds ratio (OR) and corresponding 95% confidence interval (95% CI) based on the unadjusted (single variable) and adjusted (multivariable) logistic regression models of anxiety.**

| Variable | | Anxiety | | | |
|---|---|---|---|---|---|
| | | Unadjusted | | Adjusted | |
| | | OR (95%) | P value | OR (95%) | P-value |
| **Demography** | | | | | |
| Gender | | | | | |
| | Female | 1 | | 1 | |
| | Male | 1.11 (0.93-1.32) | 0.236 | 0.72 (0.59-0.89) | 0.002 |
| Age | | | | | |
| | <13 years old | 1 | | 1 | |
| | 13-15 years old | 0.68 (0.39-1.24) | 0.188 | 0.85 (0.44-1.77) | 0.651 |
| | 16-17 years old | 1.03 (0.59-1.91) | 0.909 | 1.42 (0.73-2.96) | 0.325 |
| Grade | | | | | |
| | Grade 8-9 | 1 | | | |
| | Grade 10-11 | 1.21 (1.00-1.46) | 0.048 | | |
| | Grade 12-13 | 1.75 (1.34-2.28) | <0.001 | | |
| **Food Habits** | | | | | |
| Adequate fruit & veg intake | | | | | |
| | No | 1 | | 1 | |
| | Yes | 0.58 (0.43-0.76) | 0.001 | 0.56 (0.41-0.76) | 0.001 |
| Excess carbonate drinks | | | | | |
| | No | 1 | | | |
| | Yes | 1.20 (0.91-1.55) | 0.170 | | |
| Fast food consumption | | | | | |
| | No | 1 | | 1 | |
| | Yes | 1.62 (1.36-1.93) | <0.001 | 1.44 (1.19-1.76) | 0.001 |
| Hungry for the past 30 days | | | | | |
| | No | 1 | | 1 | |
| | Yes | 3.74 (3.04-4.59) | <0.001 | 2.58 (2.04-3.25) | <0.001 |
| **Personal Hygiene** | | | | | |
| Adequate oral health | | | | | |
| | No | 1 | | | |
| | Yes | 0.81 (0.67-0.97) | 0.023 | | |
| Adequate handwash hygiene | | | | | |
| | No | 1 | | | |
| | Yes | 0.86 (0.72-1.01) | 0.080 | | |
| **Parental Engagement** | | | | | |
| Parental understanding | | | | | |
| | No | 1 | | 1 | |
| | Yes | 0.66 (0.55-0.81) | <0.001 | 0.75 (0.60-0.95) | 0.017 |
| Parental supervision | | | | | |
| | No | 1 | | | |
| | Yes | 0.55 (0.44-0.69) | <0.001 | | |
| **Social Engagement** | | | | | |
| Close friends | | | | | |
| | No | 1 | | 1 | |
| | Yes | 0.54 (0.39-0.75) | 0.001 | 0.59 (0.39-0.86) | 0.007 |
| Peers support | | | | | |
| | No | 1 | | 1 | |
| | Yes | 1.21 (0.97-1.54) | 0.099 | 1.36 (1.04-1.78) | 0.027 |
| Physically active | | | | | |
| | No Active | 1 | | | |

| | | | | | |
|---|---|---|---|---|---|
| | Moderate Active | 0.78 (0.62-0.99) | 0.036 | | |
| | Intensive Active | 0.90 (0.71-1.14) | 0.375 | | |
| Truancy | | | | | |
| | No | 1 | | | |
| | Yes | 1.59 (1.32-1.89) | <0.001 | | |
| **Behavior** | | | | | |
| Physical fight | | | | | |
| | No | 1 | | 1 | |
| | Yes | 2.23 (1.87-2.66) | <0.001 | 1.45 (1.18-1.79) | 0.001 |
| Bullied | | | | | |
| | No | 1 | | 1 | |
| | Yes | 3.89 (3.24-4.68) | <0.001 | 2.92 (2.37-3.59) | <0.001 |
| **Substance Abuse** | | | | | |
| Alcohol | | | | | |
| | No | 1 | | | |
| | Yes | 3.00 (1.97-4.54) | <0.001 | | |
| Drugs | | | | | |
| | No | 1 | | | |
| | Yes | 1.95 (1.16-3.19) | 0.009 | | |
| Smoking status | | | | | |
| | Non-Smoker | 1 | | 1 | |
| | Smoker | 2.59 (1.79-3.72) | <0.001 | 1.39 (0.89-2.16) | 0.144 |
| | Ex-Smoker | 1.99 (1.09-3.49) | 0.020 | 1.31 (0.63-2.59) | 0.452 |
| | Unknown | 2.42 (1.71-3.39) | <0.001 | 1.89 (1.26-2.83) | 0.002 |
| Second-hand smoking | | | | | |
| | No | 1 | | 1 | |
| | Yes | 1.87 (1.57-2.23) | <0.001 | 1.27 (1.04-1.55) | 0.017 |

**Supplementary Table S4: Estimates of odds ratio (OR) and corresponding 95% confidence interval (95% CI) based on the unadjusted (single variable) and adjusted (multivariable) logistic regression models of suicidal ideation.**

| Variable | Anxiety | | | |
|---|---|---|---|---|
| | Unadjusted | | Adjusted | |
| | OR (95%) | P-value | OR (95%) | P-value |
| **Demography** | | | | |
| Gender | | | | |
| Female | 1 | | | |
| Male | 0.91 (0.62-1.31) | 0.604 | | |
| Age* | | | | |
| <13-15 years old | 1 | | | |
| 16-17 years old | 0.98 (0.03-0.05) | 0.921 | | |
| Grade | | | | |
| Grade 8-9 | 1 | | | |
| Grade 10-11 | 1.14 (0.77-1.69) | 0.521 | | |
| Grade 12-13 | 1.25 (0.67-2.20) | 0.459 | | |
| **Food Habits** | | | | |
| Adequate fruit & veg intake | | | | |
| No | 1 | | | |
| Yes | 0.92 (0.52-1.52) | 0.754 | | |
| Excess carbonate drinks | | | | |
| No | 1 | | | |
| Yes | 1.57 (0.92-2.55) | 0.076 | | |
| Fast food consumption | | | | |
| No | 1 | | 1 | |
| Yes | 1.49 (1.03-2.15) | 0.034 | 1.57 (1.06-2.31) | 0.023 |
| Hungry for the past 30 days | | | | |
| No | 1 | | | |
| Yes | 1.63 (1.03-2.51) | 0.031 | | |
| **Personal Hygiene** | | | | |
| Adequate oral health | | | | |
| No | 1 | | | |
| Yes | 0.76 (0.52-1.12) | 0.156 | | |
| Adequate handwash hygiene | | | | |
| No | 1 | | | |
| Yes | 0.73 (0.49-1.05) | 0.097 | | |
| **Parental Engagement** | | | | |
| Parental understanding | | | | |
| No | 1 | | 1 | |
| Yes | 0.29 (0.20-0.43) | <0.001 | 0.48 (0.31-0.75) | 0.001 |
| Parents Supervision | | | | |
| No | 1 | | 1 | |
| Yes | 0.24 (0.16-0.35) | <0.001 | 0.43 (0.27-0.69) | 0.001 |
| **Social Engagement** | | | | |
| Close friends | | | | |
| No | 1 | | 1 | |
| Yes | 0.45 (0.26-0.86) | 0.009 | 0.54 (0.29-1.05) | 0.053 |
| Peers support | | | | |
| No | 1 | | | |
| Yes | 0.76 (0.50-1.20) | 0.227 | | |
| Physically active | | | | |
| No activity | 1 | | 1 | |
| Moderate activity | 0.32 (0.20-0.49) | <0.001 | 0.37 (0.23-0.59) | <0.001 |

| | | | | |
|---|---|---|---|---|
| Intensive activity | 0.40 (0.26-0.62) | <0.001 | 0.56 (0.35-0.89) | 0.015 |
| Truancy | | | | |
| No | 1 | | 1 | |
| Yes | 1.69 (1.16-2.45) | 0.005 | 1.46 (0.99-2.17) | 0.056 |
| **Behavior** | | | | |
| Physical fight | | | | |
| No | 1 | | | |
| Yes | 1.48 (1.02-2.14) | 0.037 | | |
| Bullied | | | | |
| No | 1 | | 1 | |
| Yes | 2.10 (1.45-3.07) | <0.001 | 1.57 (1.06-2.31) | 0.025 |
| **Substance Abuse** | | | | |
| Alcohol | | | | |
| No | 1 | | | |
| Yes | 1.97 (0.75-4.25) | 0.119 | | |
| Drugs | | | | |
| No | 1 | | | |
| Yes | 2.38 (0.82-5.51) | 0.069 | | |
| Smoking status | | | | |
| Non-Smoker | 1 | | | |
| Smoker | 2.28 (1.05-4.38) | 0.023 | | |
| Ex-Smoker | 3.65 (1.37-8.11) | 0.004 | | |
| Unknown | 1.20 (0.46-2.56) | 0.673 | | |
| Second-hand smoking | | | | |
| No | 1 | | | |
| Yes | 1.09 (0.75-1.58) | 0.642 | | |

*There is no data for age group <13 years with suicidal ideation, so the age categories less than 13 years and 13-15 years were merged into one group
